# Ferroptosis-related Genes for Overall Survival Prediction in Patients with Colorectal Cancer can be Inhibited by Gallic acid

**DOI:** 10.7150/ijbs.57164

**Published:** 2021-03-01

**Authors:** Zongchao Hong, Peili Tang, Bo Liu, Chongwang Ran, Chong Yuan, Ying Zhang, Yi Lu, Xueyun Duan, Yanfang Yang, Hezhen Wu

**Affiliations:** 1 Faculty of Pharmacy, Hubei University of Chinese Medicine, Wuhan, China.; 2 Key Laboratory of Traditional Chinese Medicine Resources and Chemistry of Hubei Province, Wuhan, China.; 3 Collaborative Innovation Center of Traditional Chinese Medicine of New Products for Geriatrics Hubei Province, Wuhan, China.; 4 Hubei Provincial Hospital of Traditional Chinese Medicine, Wuhan, China.

**Keywords:** Ferroptosis, Colorectal cancer, Gallic acid, Natural product.

## Abstract

Colorectal cancer (CRC) is one of the most deadly malignant tumors, which seriously threatens human health. Ferroptosis, a new type of iron-dependent cell regulatory necrosis. Inducing ferroptosis of tumor cells is regarded as a potential treatment strategy. However, the prognostic value of ferroptosis-related genes in CRC remains to be further elucidated. Gallic acid, widely used in the chemical, pharmaceutical, and food fields, is a dietary supplement with potential prescription significance. In this study, the mRNA expression profiles and corresponding clinical data of CRC patients were downloaded from public databases. Gene Expression Profiling Interactive Analysis (GEPIA) was used to evaluate the expression levels of ferroptosis-related genes. In addition, bioinformatics analysis showed the prognostic value of ferroptosis-related genes in CRC. Molecular docking predicts the binding status of gallic acid and ferroptosis-related genes. The experiment confirmed the correctness of the predicted results. Our results show that in the TCGA cohort, 30 ferroptosis-related genes are differentially expressed between CRC and adjacent normal tissues. Among them, eight differentially expressed genes are related to overall survival. Gallic acid can bind to ferroptosis-related targets and regulate the expression of corresponding proteins, and ferroptosis inhibitors reversed the experimental results. In summary, eight new ferroptosis-related genes can be used to predict the prognosis of CRC. Gallic acid can improve CRC by regulating ferroptosis.

## 1. Introduction

Ferroptosis is a newly-discovered form of cell death proposed in recent years, which is different from other death patterns in morphology, biochemical characteristics, and regulatory mechanisms. Ferroptosis relies on the generation of reactive oxygen species (ROS) and the severe lipid peroxidation of iron overload. It is characterized by increased iron and ROS content, decreased cysteine uptake, and increased release of arachidonic acid-mediated factors 1. Mitochondria is indispensable in iron metabolism, material, and energy metabolism, as it is the main organelle of iron utilization, catabolism, and anabolic pathways. More importantly, mitochondria are essential for lipid metabolism and maintaining iron homeostasis 2. Ferroptosis is the link between metabolism, redox biology, and human health. Increasingly evidence shows that ferroptosis may be caused in cancer treatment, especially in the elimination of aggressive malignant tumors that are resistant to traditional therapies 3. However, activation of ferroptosis will accelerate neurodegenerative diseases, including Parkinson's disease (PD) and Alzheimer's disease 4. Obviously, activating or inhibiting ferroptosis can be used to deal with various cancers and neurodegenerative diseases and may achieve satisfactory therapeutic effects.

Colorectal cancer (CRC) is a common form of malignant tumor in gastrointestinal tract cancer, and the symptoms in the early stages are unobservable. With the increase of cancer, the patients defecation pattern change, blood in the stool, diarrhea, alternating diarrhea and constipation, and other clinical symptoms. Colorectal cancer is often first diagnosed at an advanced stage with systemic symptoms such as anemia and weight loss. The incidence and mortality of CRC are second only to gastric cancer, esophageal cancer, and primary liver cancer 5-6. The number of new cases and deaths of CRC continues to increase, and the global burden of colorectal cancer worldwide will increase significantly in the near future, and chronic colitis will be responsible for a significant increase in CRC incidence 7-8. The etiology and pathogenesis of CRC mainly considered related to metabolic changes, high fat and low cellulose diet, chronic inflammation of the colon, colon polyps, familial inheritance and other factors. Controlling these potential factors may help reduce the incidence of CRC 9-10. Many natural products have a variety of biological activities, and finding drugs to treat diseases from natural products has received increasing recognition. The active products screened out from edible plants have great utility value.

Gallic acid (also known as 3,4,5-trihydroxybenzoic acid, Figure 7I), widely found in tea leaves, oak bark, gallnut, and other plants, and appears in foods or spices such as cloves, vinegar, and fruits (strawberries, grapes, bananas, etc.) 11-12. Gallic acid has a wide range of applications in the fields of food, biology, medicine, and chemicals 13. Based on a wide range of biological activities, coupled with being almost non-toxic to the human body, gallic acid is used as a dietary supplement or additive with clinical significance. Direct consumption of vegetables and fruits containing gallic acid may also be beneficial to health. In the past few decades, more and more evidence shows that gallic acid has the effects of anti-inflammatory 14, anti-oxidant 15, and anti-viral 16. In addition, as a polyphenolic compound, gallic acid's cardioprotective and neuroprotective properties have also been discovered 17. More interestingly, gallic acid also seems to have an inhibitory effect on tumors 18. The biological activity of gallic acid depends on certain factors, such as the concentration of gallic acid, the amount of iron (Fe^3+^) in the system, and the pH of the reaction medium, and its antioxidant or pro-oxidant properties affect the reactive oxygen species (ROS) signal network, this seems to be related to mitochondria 14. However, how the biological activity of gallic acid is related to iron concentration and mitochondria is unclear.

Ferroptosis, as a new form of cell death, is of great significance in the diagnosis and treatment of tumors. However, whether ferroptosis-related genes are related to the prognosis of CRC patients remains unclear. Whether the anti-tumor activity of gallic acid is related to ferroptosis also needs to be further clarified. In this study, we first downloaded the mRNA expression profile of CRC patients and the corresponding clinical data from a public database. Then, we analyzed the prognostic value of ferroptosis-related differentially expressed genes (DEGs) in the TCGA cohort, and verified it in the GEO cohort. Finally, we investigated the regulatory effect of gallic acid on ferroptosis in CRC.

## 2. Materials and Methods

### 2.1. Data collection

The mRNA (RNA-seq) data of 480 cases of colon cancer patients and 41 cases of adjacent tissues have been downloaded from the TCGA website (https://portal.gdc.cancer.). The dataset (GSE32323) containing the mRNA data of 17 cases of colon cancer patients and 17 cases of adjacent tissues has been downloaded from the GEO database (https://www.ncbi.nlm.nih.gov/gds/). The obtained cohort were normalized using the Omicshare (https://www.omicshare.com/tools/index.php/) online system. Both TCGA and GEO data are public. Therefore, this study exempted the approval of the local ethics committee. The current research follows TCGA and GEO data access policies and publication guidelines.

GeneCards (https://www.Genecards.org/) is a comprehensive searchable database. We searched GeneCards with "ferroptosis" as the key word and obtained 103 target genes related to ferroptosis and are provided in Supplementary Table S1.

### 2.2. Identification of differentially expressed genes (DEGs)

The R statistical package software EdgeR (Empirical analysis of Digital Gene Expression in R, http://www.bioconductor.org/packages/2.12/bioc/html/edgeR.html) was used to assess differential expression. *p*-values were adjusted using the false discovery rate (FDR) method. Genes with adjusted *p* <0.05 and |log_2_ fold change (FC)| >1.0 were considered as DEGs in the combined analysis. Then, we take the intersection of the DEGs and ferroptosis-related genes. Obtain the differentially expressed genes related to ferroptosis in CRC.

### 2.3. Protein-protein interaction (PPI) network construction

STRING database (https://string-db.org/) uses document content management to extract protein-protein interaction relationships derived from experimental data. In addition, the STRING database also stores some calculated and predicted interaction relationships 19. As a widely used online tool, we introduced the target gene into STRING to construct PPI networks.

### 2.4. Enrichment analysis

DAVID (https://david.ncifcrf.gov/) is a biological information database and an online analysis software that provides systematic and comprehensive biological function annotation information for large-scale gene or protein lists 20. At present, the DAVID database is mainly used for the analysis of differential gene function and pathway enrichment. We utilized the DAVID database to conduct functional enrichment analysis (GO and KEGG pathway analysis) for ferroptosis-related targets. In addition, the results obtained are visualized by Hiplot (https://hiplot.com.cn/basic).

### 2.5. GEPIA-based DEGs confirmation and overall survival analysis

The GEPIA server (http://gepia.cancer-pku.cn) has been running for two years and processed ~280,000 analysis requests for ~110,000 users from 42 countries. Users can perform all expression analyses such as survival analysis and differential analysis at the isoform level. Usage of different transcripts across all cancer types can also be profiled 21. In this study, we used GEPIA to perform differential expression analysis and overall survival analysis of candidate proteins. In addition, the genes have been further verified in the GEO dataset.

### 2.6. Gene Set Enrichment Analysis (GSEA)

GSEA is an analysis method for genome-wide expression profile chip data. It compares genes with a predefined gene set (usually from functional annotations or the results of previous experiments), and then checks whether the predefined gene set is in this ranking table enrichment at the top or bottom. In this study, we introduced the genes to be analyzed by GSEA (http://www.gsea-msigdb.org/gsea/) software for enrichment analysis.

### 2.7. Molecular Docking

Download the ".sdf" format structure of gallic acid from The PubChem Project (https://pubchem.ncbi.nlm.nih.gov/). Subsequently, the structures of proteins were downloaded from the RSCB PDB database (https://www.rcsb.org/). DiscoveryStudio software was used for solvent molecules and ligand removal, hydrogenation, electron addition, and other operations. Molecular docking was then performed (Discovery Studio 2016; BIOVIA; San Diego, USA).

### 2.8. Reagents and materials

Gallic acid (#110831-201204), purchased from National Institutes for Food and Drug Control of China. ATF4 antibody (#10835-1-AP) was purchased from proteintech group, Inc (Wuhan, China). GPX4 antibody (#bs-3884R), SLC7A11 antibody (#bs-5111), and TFR1 antibody (#bs-9894R) was purchased from Beijing boosen biotechnology Co., Ltd (Beijing, China). Anti-rabbit IgG (#ab150077), iron assay kit (#ab83366) was purchased from Abcam Technology (Abcam, Cambridge, UK). GAPDH antibody (#2118) was purchased from Cell Signaling Technology (CST, USA). Ferrostatin-1 (#HY-100579) was purchased from MedChemExpress (New Jersey, USA). Reactive oxygen species detection kit (#CA1410), reduced glutathione content detection kit (#BC1175), malondialdehyde (MDA) content detection kit (#BC0025), BCA protein concentration determination kit (#PC0020) purchased from Beijing solarbio science & technology co.,ltd. (Beijing, China). CCk-8 kit (#CK04) was purchased from Dojindo (Japan). Trypsin-EDTA (#GNM25200), PBS (#GNM20012), D-Hanks (#GNM14170) were purchased from Gino Biomedical Technology Co., Ltd. (Hangzhou, China); Crystal Violet Stain (#AS1086) was purchased from Wuhan Aspen Biotechnology Co., Ltd. (Wuhan, China); 5% TNBS and pentobarbital sodium were purchased from Sigma Aldrich (USA); Mesalazine granules (#170904) were purchased from the France Aifa Pharmaceutical group.

### 2.9. Cell culture

The HCT116 cell lines were provided by the Cell Bank of the Chinese Academy of Sciences (Shanghai, China), Caco-2 cell lines were purchased from Procell Life Science&Technology Co.,Ltd. (Wuhan, China). The cell lines were cultured in DMEM medium containing 10% FBS (Grand Island, NY) and 1% penicillin-streptomycin. The culture environment was 37°C, 5% CO2 humid environment.

### 2.10. Kit detection

CCK-8 kit was used to detect cell viability, reactive oxygen kit to detect the production of ROS, GSH kit to detect the content of reduced glutathione, iron detection kit to detect Fe^2+^ content, and MDA kit to detect the degree of lipid peroxidation. All kits were used according to the instructions of the corresponding kit manufacturer.

### 2.11. Clone formation experiment

Cells in good condition were treated with 0.25% trypsin-EDTA trypsin and resuspended in 1 mL medium. After counting, the cells inoculate a 6-well plate with an inoculum of about 500 cells per well, and place it in a 37°C, 5% CO2 incubator. After the cells grow adherently, they are treated with drugs for 24 hours and cultured for 2 weeks. The cloned cells were fixed with 4% paraformaldehyde, stained with crystal violet dye at room temperature, and images were collected after drying. Cloning rate = (number of clones/number of inoculated cells) × 100%.

### 2.12. Western blot (WB)

The cells were collected and washed twice with PBS. Cells were lysed to obtain protein, and the protein concentration was determined by BCA kit. Adjust the protein concentration of each group to 2 μg/μL with 5× loading buffer and heat at 95°C for 5 minutes. Take 20 μg sample and separate it on 10% SDS-PAGE separation gel. After electrophoresis and membrane transfer, it was blocked with 5% skimmed milk powder for 1.5 hours. Incubate the primary antibody and overnight at 4°C. Wash three times with TBST buffer, and then incubate the secondary antibody for 1 hour at room temperature. After the antibody incubation was completed, wash 3 times with TBST buffer. Finally, ECL was used to develop and expose the image.

### 2.13. Animal experiment

Ulcerative colitis (UC) is usually the cause of CRC. Controlling or alleviating the occurrence and development of UC may reduce the incidence of CRC. We also want to know whether gallic acid can improve UC. Therefore, we conducted preliminary experiments on rats. Animal experiments were carried out under standard laboratory conditions in the Experimental Animal Center of Hubei Provincial Hospital of Traditional Chinese Medicine (HPHTCM, Wuhan, China). The animals were kept in a captive environment with a temperature of 25±2℃ and a relative humidity of 50-55% and a 12h light/dark cycle and acclimatized to the environment for at least one week before animal experiments. Animal welfare and experimental procedures strictly follow the guidelines of the HPHTCM Institute of Animal Research Committee (No. HBZYY-2018-043).

The UC model was established as described previously 22. In short, we randomly selected 3 rats as the normal group and then induced the UC model with TNBS for the remaining rats. The successfully induced UC models were divided into model group, positive control group (Mesalazine as positive drug), and gallic acid group. The positive control group was administered with 0.5 g/kg/d of mesalazine, and the gallic acid group was administered with 10 mg/kg/d of gallic acid by gavage. The normal group and model group received the same amount of saline (1 ml/d). After 14 days of continuous administration, two rats from each group were randomly selected, and their colonic tissues were separated for pathological examination after euthanizing.

### 2.14. Statistical analysis

Student's t-test was used to compare gene expression between tumor tissues and adjacent nontumorous tissues. Graphpad Prism 7.0 was used to perform data analysis. The statistical value p<0.05 indicates that the difference is statistically significant.

## 3. Results

### 3.1. Screening of ferroptosis-related DEGs by combined analysis

**Figure 1:**
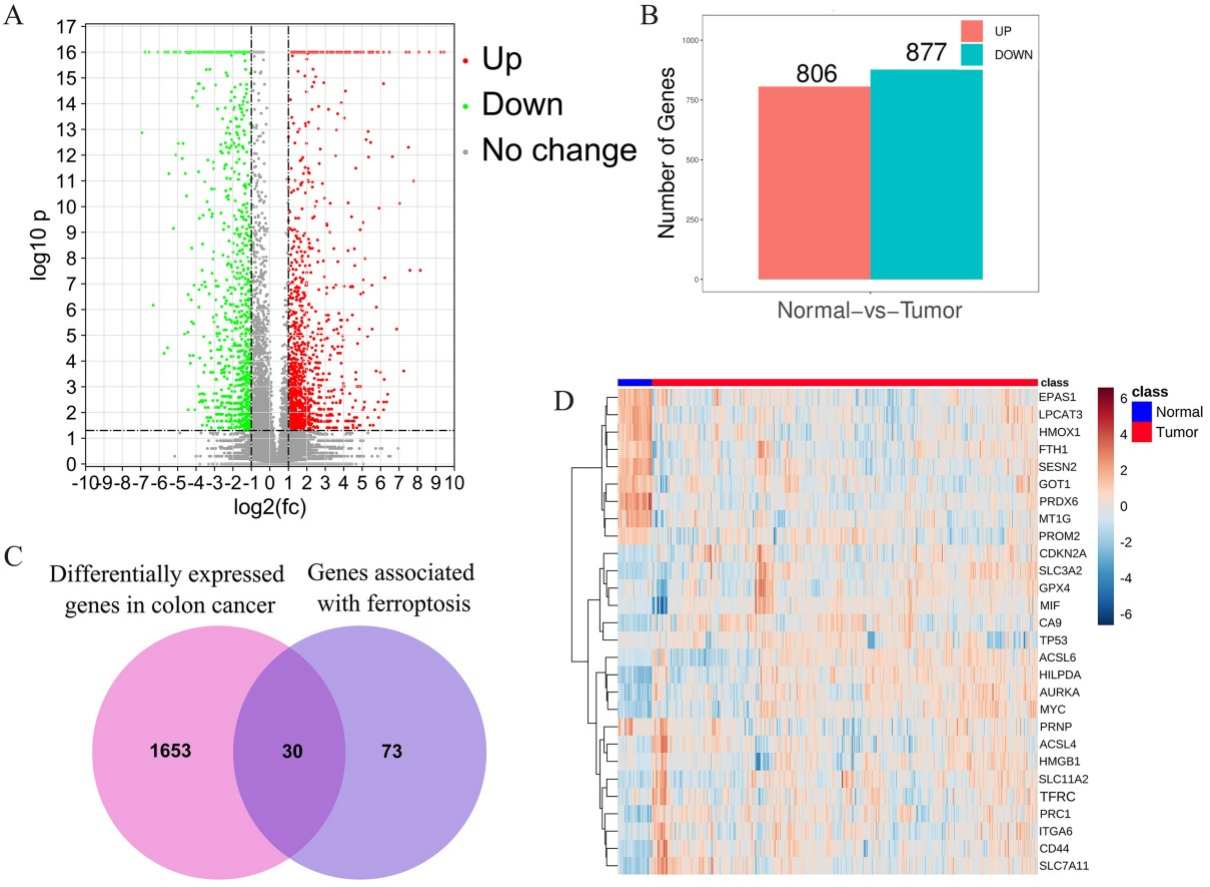
** Identification of the candidate ferroptosis-related genes in the TCGA cohort**. A.Volcano plots, used to identify differentially expressed genes between tumors and normal tissues; B.Statistics on the results of volcano plots; C.Venn diagram to identify ferroptosis-related differentially expressed genes between tumor and adjacent normal tissue; D. The expression trend of 30 overlapping genes in tumor tissues.

After combined analysis, 1683 DEGs (806 up-regulated and 877 down-regulated, displayed in supplementary table S2) were identified and are shown in volcano plots (Figure 1A,B). The polarity of genes described as “up-regulated” or “down-regulated” in this article is with respect to tumor vs. normal. Among the 1683 DEGs, 30 genes are related to ferroptosis (Figure 1C). The expression of these 30 genes in the TCGA data set (including 480 cancerous tissues and 41 para cancerous tissues) is shown in the heatmap (Figure 1D). The top five up-regulated genes were CA9, ACSL6, CDKN2, SLC7A11, and HILPDA, whereas the top five down-regulated genes were MT1G, HMOX1, PRDX6, LPCAT3, and EPAS1 (Shown in supplementary table S3).

### 3.2. PPI network construction and functional enrichment analysis of DEGs

**Figure 2:**
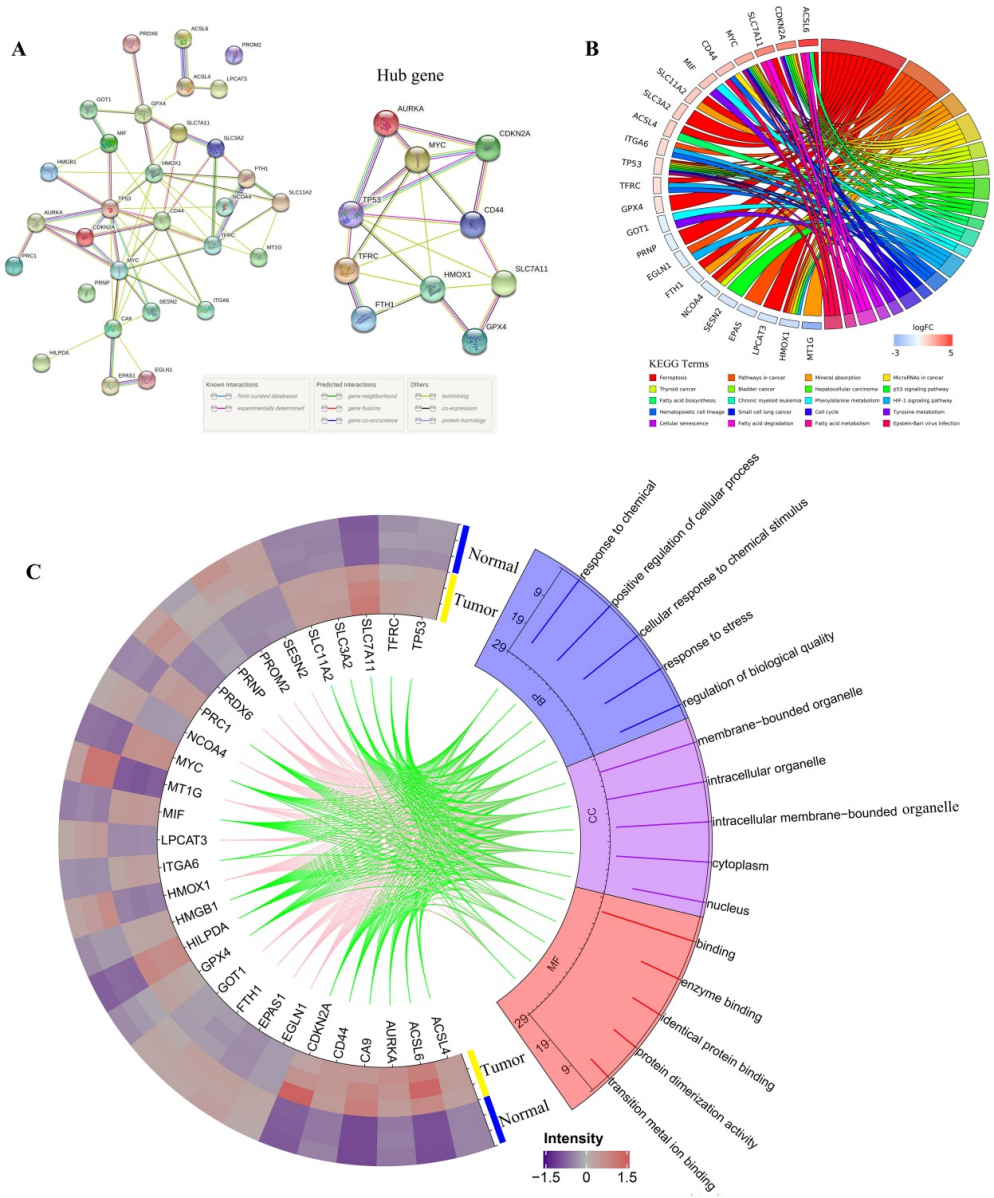
** PPI network,Gene Ontology (GO) and Kyoto Encyclopedia of Genes and Genomes (KEGG) analyses of all differentially expressed genes related to ferroptosis.** A. PPI network constructed by STRING; B. KEGG pathways; C. Go analysis items, the green line indicates the high expression of the corresponding gene in CRC, and the pink line indicates the low expression of the corresponding gene in CRC.

All DEGs were imported to STRING to perform PPI network construction. The interaction network among these genes indicated that GPX4, SLC7A11, CDKN2A, TP53, AURKA, HMOX1, TFRC, FIH1, MYC, and CD44 may be the hub genes (Figure 2A). Then, all 30 DEGs were utilized to perform GO and KEGG analyses. Go analysis included biological process (BP), cellular component (CC), and molecular function (MF), the enriched entries are shown in Supplementary Table S4. As shown in Figure 2C, the top five terms BP for GO analysis were response to chemical, positive regulation of cellular process, cellular response to chemical stimulus, response to stress, and regulation of biological quality. The top five CC terms for GO analysis were membrane-bounded organelle, intracellular organelle, intracellular membrane-bounded organelle, cytoplasm, and nucleus. The top five MF terms for GO analysis were binding, enzyme binding, identical protein binding, protein dimerization activity, and transition metal ion binding. For KEGG pathway analysis, DEGs were mostly enriched in ferroptosis, pathways in cancer, mineral absorption, microRNAs in cancer, and thyroid cancer (Figure 2B).

### 3.3. Identification and overall survival analysis of ferroptosis related DEGs

**Figure 3:**
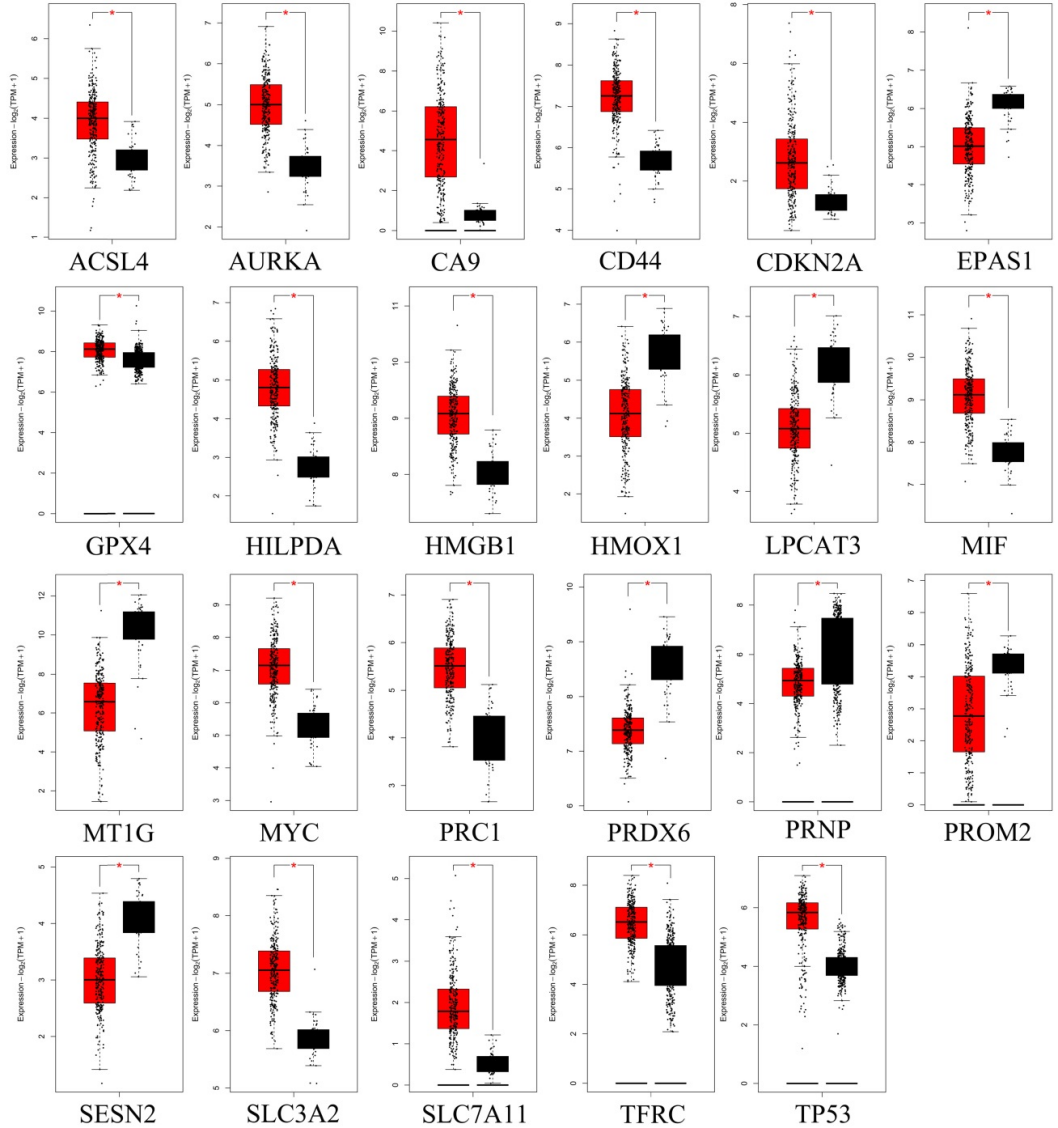
GEPIA analyzed genes that are significantly differentially expressed in CRC. Red color represents tumor tissue, and black color represents normal tissue.

**Figure 4:**
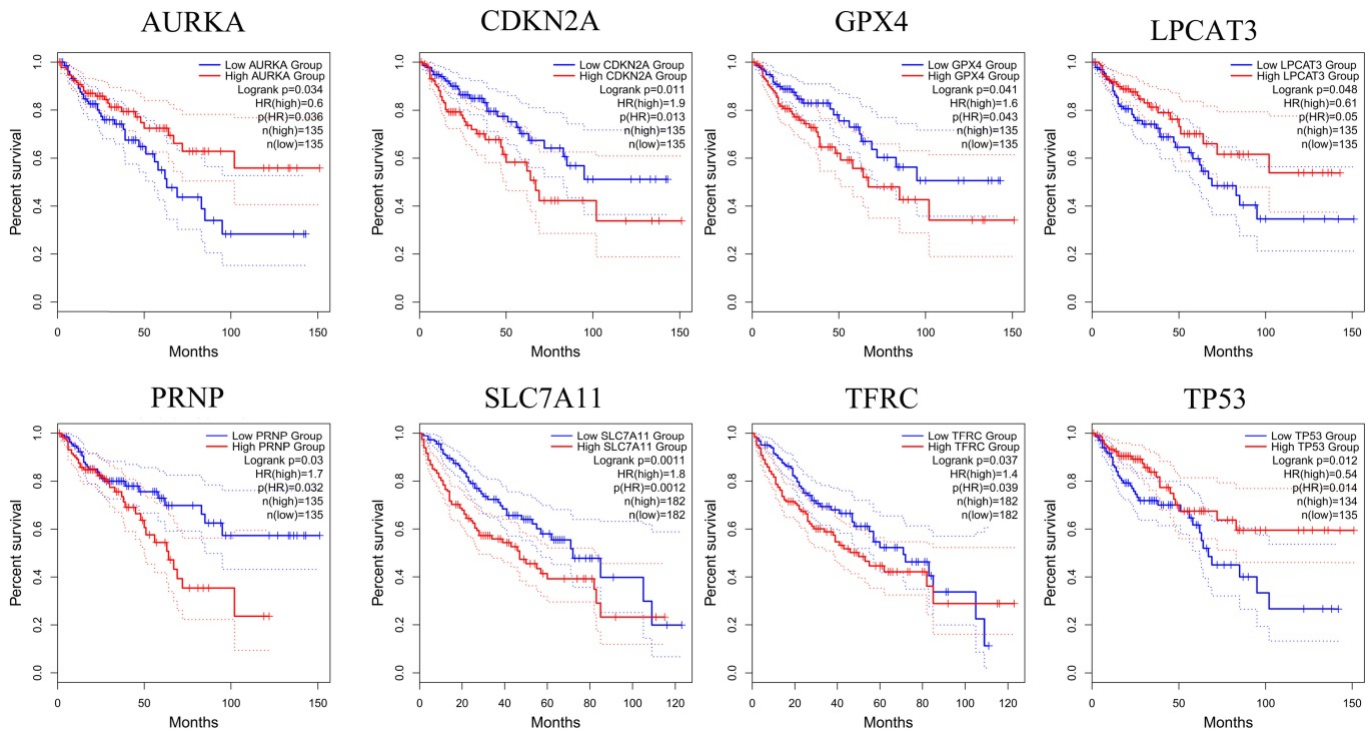
Genes analyzed by GEPIA that affect the overall survival of CRC patients. The red line indicates the high expression group of genes and the blue line represents the low expression group of genes.

**Figure 5:**
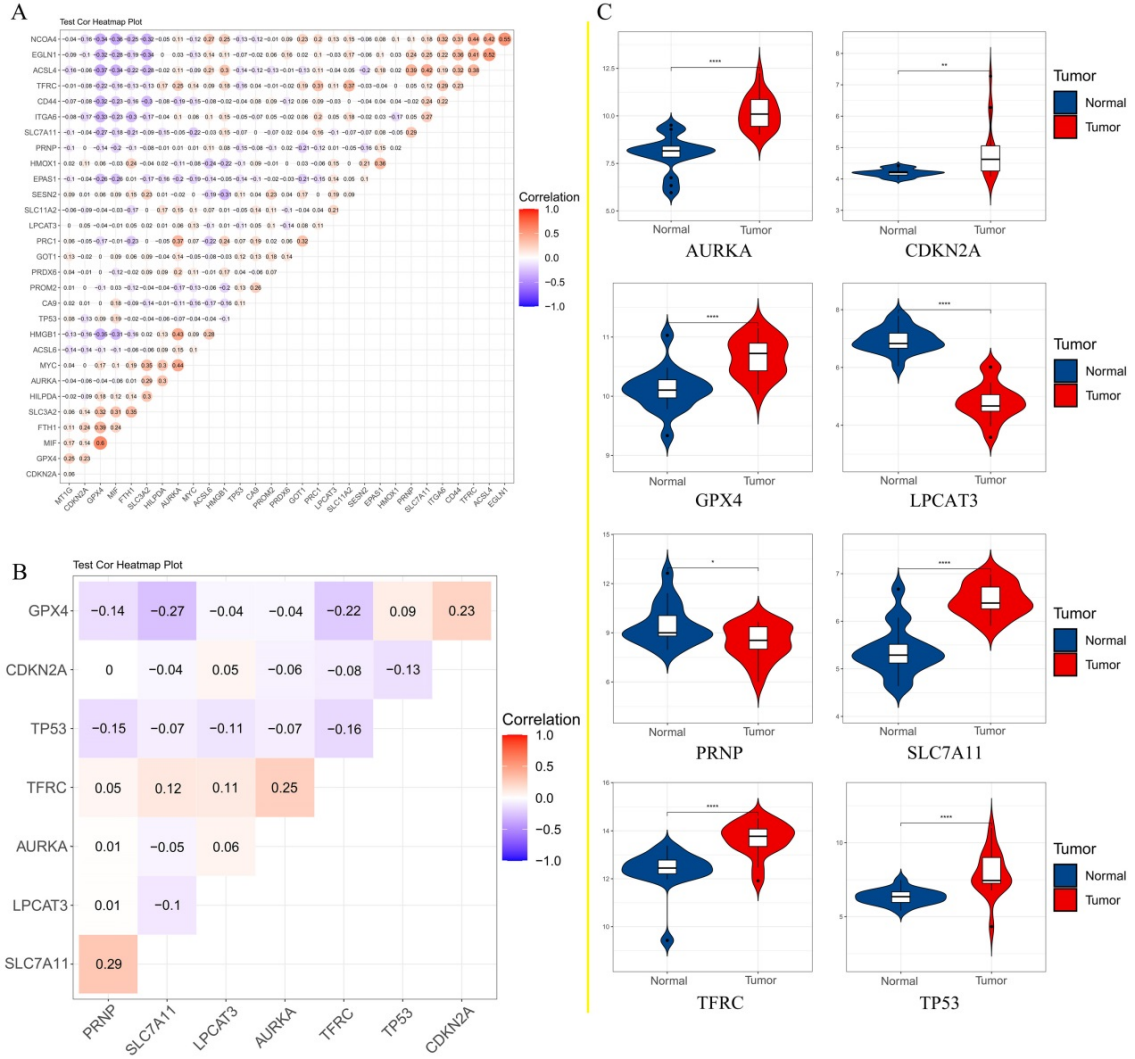
A. Correlation analysis of 30 genes; B. Correlation analysis of 8 genes affecting the overall survival of patients; C. The expression of 8 genes that affect the overall survival of patients in the GEO data set.

To further confirm the accuracy of the analysis of DEGs, we input 30 ferroptosis-related genes into the GEPIA server for in-depth analysis. Figure 3 shows the analysis results. Among the 30 genes, 23 genes were confirmed to have significant differential expression in CRC. According to the correlation analysis of gene coexpression (Figure 5A), all 30 genes were correlated with each other. Among them, MIF and GPX4 (correlation=0.6), NCOA4 and EGLN1 (correlation=0.55), ACSL4 and TFRC (correlation=0.52), MYC and AURKA (correlation=0.44), NCOA4 and TFRC (correlation=0.44) were the five pairs of genes with the most positive correlation. However, ACSL4 and GPX4 (correlation=-0.37), NCOA4 and MIF (correlation=-0.36), HMGB1 and GPX4 (correlation=-0.35), EGLN1 and SLC4A2 (correlation=-0.34), ACSL4 and MIF (correlation=-0.34) were the five most negatively correlated genes. In addition, the results of GEPIA analysis showed that the low expression of AURKA, LPCAT3, and TP53 and the high expression of CDKN2A, GPX4, PRNP, SLC7A11, and TFRC are more likely to encounter CRC patients death earlier and shorten survival time (Figure 4, *p*<0.05). Moreover, the differential expression of these 8 genes has been further verified in the GEO data set (Figure 5C). A deeper understanding is that these 8 genes may provide insight into the prognosis of CRC. Certainly, there is a positive correlation between SLC7A11 and PRNP (correlation=0.25), TFRC and AURKA (correlation=0.25), GPX4 and CDKN2A (correlation=0.23), and TFRC and SLC7A11 (correlation=0.12). Contrary, SLC7A11 and GPX4 (correlation=-0.27), TFRC and GPX4 (correlation=-0.22), TP53 and TFRC (correlation=-0.16), CDKN2A and TP53 (correlation=-0.13) have a negative correlation (Figure 5B).

### 3.4. GSEA reveals the potential biological functions of 8 genes

**Figure 6:**
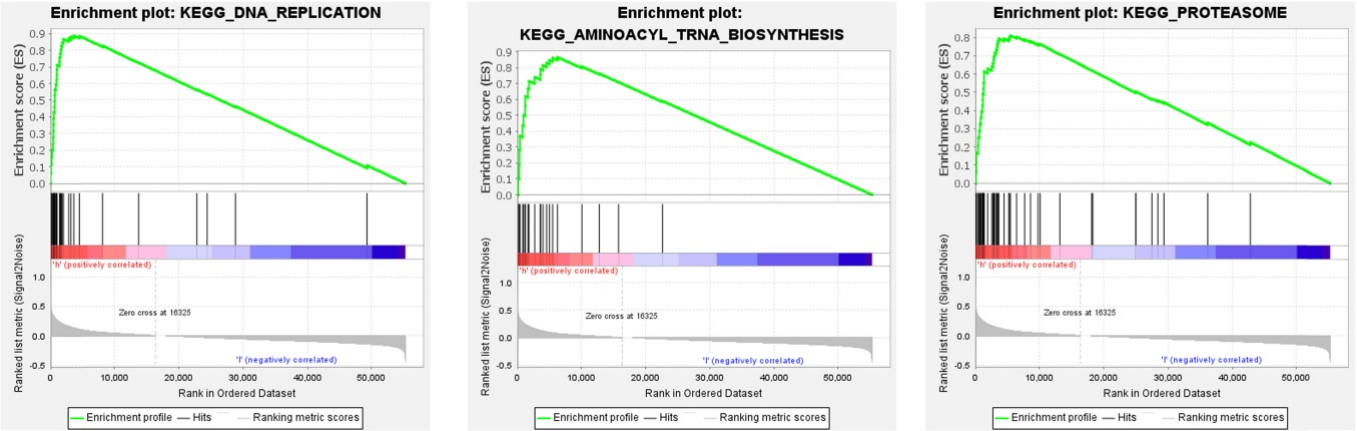
The top three GSEA terms, according to normalized enrichment scores in the high-expression group of genes.

We performed GSEA on 8 genes related to the overall survival of CRC patients. According to the standardized enrichment scores, these genes in the highly expressed gene set are mainly related to aminoacyl tRNA biosynthesis, DNA replication, and proteasome (Figure 6).

### 3.5. Gallic acid has the potential to regulate ferroptosis

**Figure 7:**
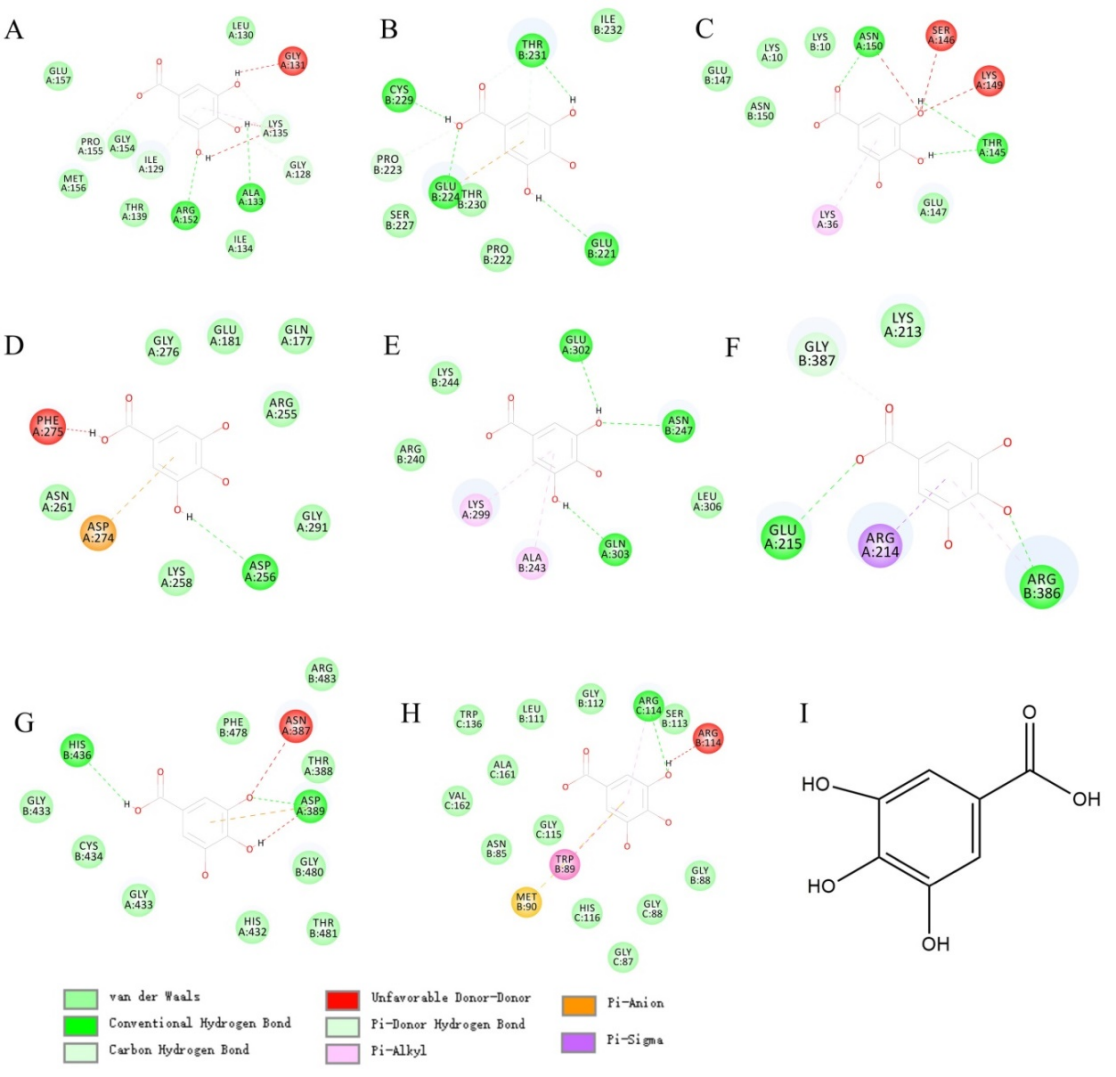
** The molecular docking between gallic acid and corresponding target.** A. GPX4(6hkq)-Gallic acid; B. TP53(3zme)-Gallic acid; C. TRFC(6y76)-Gallic acid; D. AURKA(3p9j)-Gallic acid; E. ATF4(1CI6)-Gallic acid; F. HSPA5(3ldl)-Gallic acid; G. NRF2(3zgc)-Gallic acid; H. OPRS1(6djz)-Gallic acid; I.Gallic acid.

In view of the wide application and pharmacological activity of gallic acid. We virtual docked gallic acid with 8 targets related to CRC overall survival. Surprisingly, gallic acid has the potential to bind GPX4, TP53, TRFC, and AURKA (Figures 7A, B, C, D; detailed docking scores and binding forms are shown in Table 1). This has to make us predict that gallic acid can regulate the ferroptosis process in CRC.

### 3.6. Gallic acid can inhibit the growth of colon cancer cells

**Figure 8:**
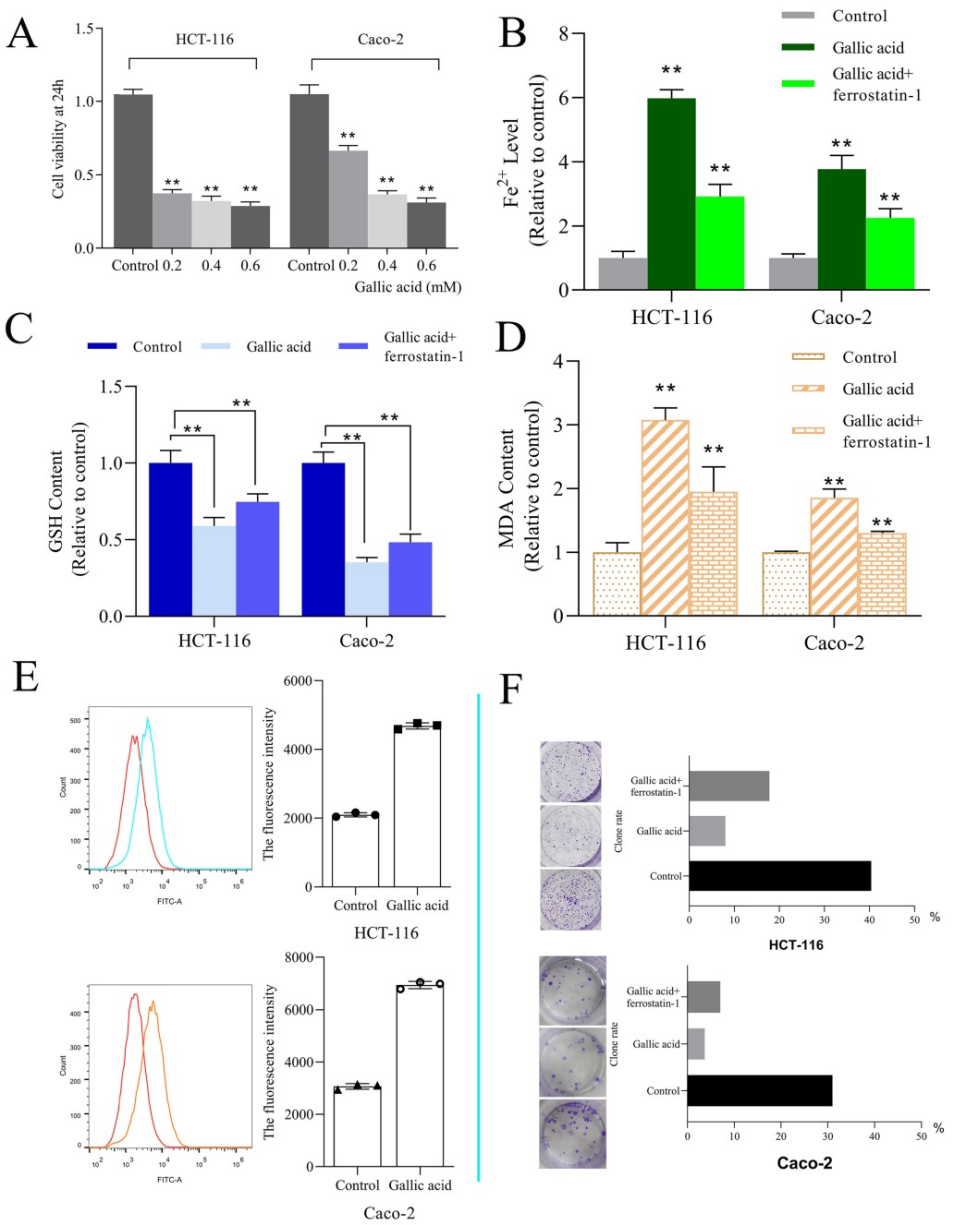
A. CCK-8 was used to investigate the fine inhibitory ability of gallic acid on HCT116 cell lines; B. Gallic acid treatment caused changes in the content of Fe^2+^; C. Gallic acid treatment caused changes in the content of GSH; D. Gallic acid treatment caused changes in the content of MDA; E. ROS production after gallic acid treatment; F. Influence of gallic acid treatment on clone formation. Ferrostatin-1 is a ferroptosis inhibitor, which reverses the trend of changes caused by gallic acid. **p < 0.01, *p < 0.05.

HCT-116 and Caco-2 cell lines were used to determine the bioactivity of gallic acid. Different concentrations of gallic acid (0.2, 0.4, or 0.6 mM) significantly reduced cell viability in a dose-dependent manner (Figure 8A). Since the use of 0.2 mM gallic acid led to a good inhibitory effect, this concentration was used for subsequent experiments. Furthermore, we performed clone formation experiments on HCT116 and Caco-2 cells, and ferrostatin-1 (a ferroptosis inhibitor with a dose of 2 μM) was used as a control. We found that the clone formation rate of the control group without any drug treatment was 40.40%, the clone formation rate of the gallic acid group was 8.0%, and the clone formation rate of the gallic acid combined with ferrostatin-1 group was 17.80%. The same phenomenon in Caco-2 cells has also been confirmed (Figure 8F). These results indicate that gallic acid has an inhibitory effect on the growth of colon cancer cells, and ferroptosis inhibitors can reverse this phenomenon.

### 3.7. The intervention of gallic acid will cause the increase of ROS, MDA, Fe^2+^, and the consumption of GSH

ROS and iron accumulation, glutathione depletion, and lipid peroxidation are characteristics of ferroptosis 23. Therefore we tested the changes in these indicators after the intervention of gallic acid. The results showed that after the gallic acid treatment, Fe^2+^ (Figure 8B), ROS (Figure 8E), and MDA (Figure 8D) were significantly increased compared to the control group, and at the same time, the content of GSH was significantly reduced (Figure 8C). There is no dispute that the intervention of ferrostatin-1 while treating the cells with gallic acid reversed the changes in these indicators to varying degrees. This makes us more convinced that gallic acid can induce ferroptosis in HCT116 cells and Caco-2 cells.

### 3.8. Gallic acid inhibits the expression of key ferroptosis proteins

**Figure 9:**
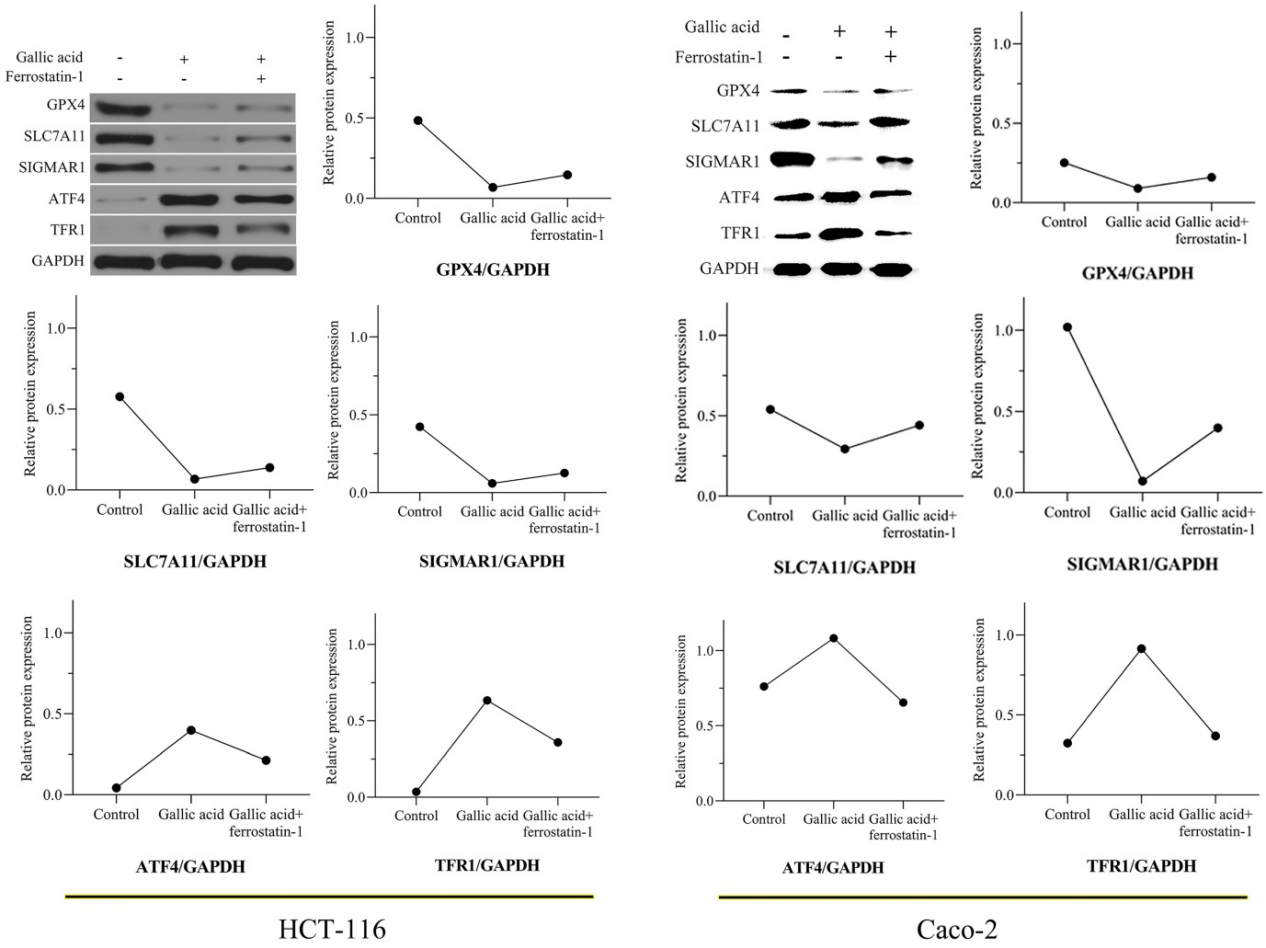
Gallic acid affects the expression of ferroptosis-related proteins. GAPDH serves as an internal reference protein. The relative expression levels of GPX4, SLC7A11, and SIGMAR1 decreased, while the relative expression levels of ATF4 and TFR1 increased. Ferrostatin-1 as a ferroptosis inhibitor reversed the expression trend of these proteins.

In view of the positive significance of GPX4, SLC7A11, and TFRC (also called TFR1) in the treatment of CRC, we tested whether the expression of these proteins changes during the intervention of gallic acid through WB experiments. After 24 hours of treatment with gallic acid, the expression of GPX4 and SCL7A11 was significantly inhibited, while the expression of TFR1 was significantly increased. Consistent with the expected results, the ferroptosis inhibitor ferrostatin-1 was given to the gallic acid treatment at the same time, and the expression of these proteins was reversed, although it did not return to the same level as the control group. In addition, we also tested the protein expression of SIGMAR1 and ATF4. Consistent with the molecular docking results in Table 1. Gallic acid inhibits the expression of SIGMAR1 and activates ATF4. Similarly, ferrostatin-1 reverses these trends. These results are shown in Figure 9. Based on the above experimental results, we believe that the inhibitory effect of gallic acid on CRC is related to the expression of GPX4, SLC7A11, SIGMAR1, ATF4, and TFR1. The further result is that the reason that gallic acid inhibits CRC is related to ferroptosis.

### 3.9. Gallic acid can improve the progression of UC

**Figure 10:**
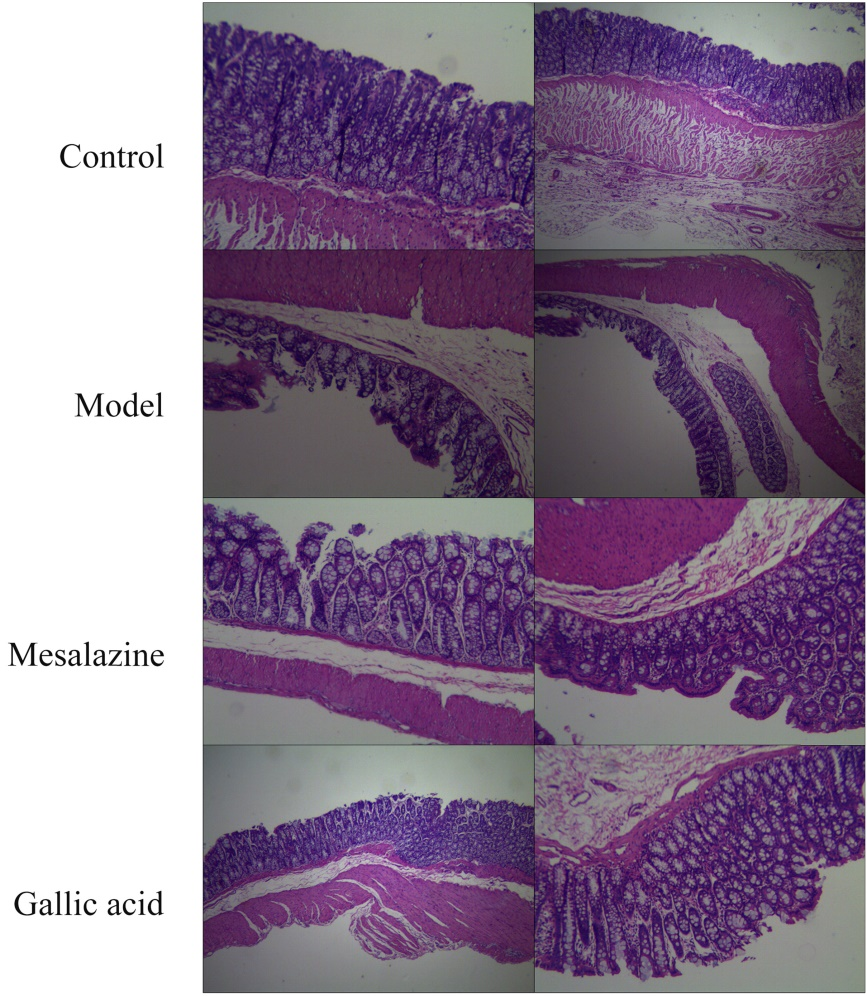
Pathological changes of colonic mucosa(HE,×40). A. Normal group; B. Model group; C. Mesalazine positive control group; D. Gallic acid group.

As shown in Figure 10, the colon structure of the normal group was clear, and the mucosal epithelium was intact. In contrast, the colon structure in the model group was destroyed, with multiple mucosal erosions, punctate necrosis, and bleeding. The mucosa of the positive control group was intact and resembled a newborn mucosa. The colon of the gallic acid group was similar to the positive control group. So the result of preliminary research is that gallic acid can improve the progress of UC, but more research is needed to reveal how gallic acid can alleviate the progress of UC. In any case, this provides a more experimental basis for gallic acid to become a dietary supplement with prescription significance.

## 4. Discussion

At the beginning of the study, we predicted the DEGs associated with ferroptosis in CRC and obtained 8 key genes that affect the overall survival of patients. Among these genes, GPX4 prevents ferroptosis in cells by inhibiting the accumulation of lipid peroxides in the cells. When GPX4 is inhibited, it can lead to the accumulation of intracellular ROS and induce ferroptosis 24. SLC7A11 is a cystine/glutamate xCT transporter that can control the production of phenylalanine pigment. At present, a lot of evidence shows that SLC7A11 promotes the uptake of cystine and the biosynthesis of glutathione, thereby preventing oxidative stress and ferroptosis of cells 25. Interestingly, ATF4 is one of the important media to maintain metabolic stability and oxidative stress balance in cells. In cells with ferroptosis, the expression of ATF4 and GPX4 are usually negatively correlated, and this result has also been confirmed in our experiments. Although the regulatory relationship between the enhancement of ATF4 activity and GPX4 is not very clear, ATF4 can regulate the occurrence of ferroptosis by regulating the expression of GPX4 26. In addition, TFR1 is a key protein in cellular iron metabolism and is essential for iron transport 27.

Gallic acid is widely found in edible plants and can be used as a food additive in food. It is currently widely used in food, medicine, and chemical industries. Gallic acid is a polyphenolic compound with a variety of biological activities (anti-inflammatory, antibacterial, neuroprotective, etc.). It is worth noting that gallic acid is almost non-toxic when taken orally (No toxic effect was shown at 5000 mg/kg body weight) 28. However, although its toxicity to normal cells is almost negligible, it is known for its selective cytotoxicity to cancer cells, which indicates that it is a valuable food additive and a potential nutritional supplement to prevent cancer risk 29.

CRC is a multifactorial disease related to diet, the improvement in the incidence and mortality of CRC is considered the result of changes in CRC risk factor patterns, cancer prevention, early diagnosis through screening, and better treatment. Many studies have found that eating more fruits and vegetables is associated with a decrease in cancer incidence 30. This discovery has stimulated people's interest in screening for the potential role of various chemical components in fruits and vegetables and general plants in controlling or preventing cancer. At present, the widespread use of various plants and their extracts in traditional medicine worldwide has shifted the focus of research toward exploring the beneficial effects of various plant components, especially phytochemicals, in various human health conditions including cancer 31. In addition, a large amount of literature has reported that many phytochemicals have shown anti-cancer effects in various *in vitro* and *in vivo* cancer models. Gallic acid is one of these phytochemicals.

**Figure 11:**
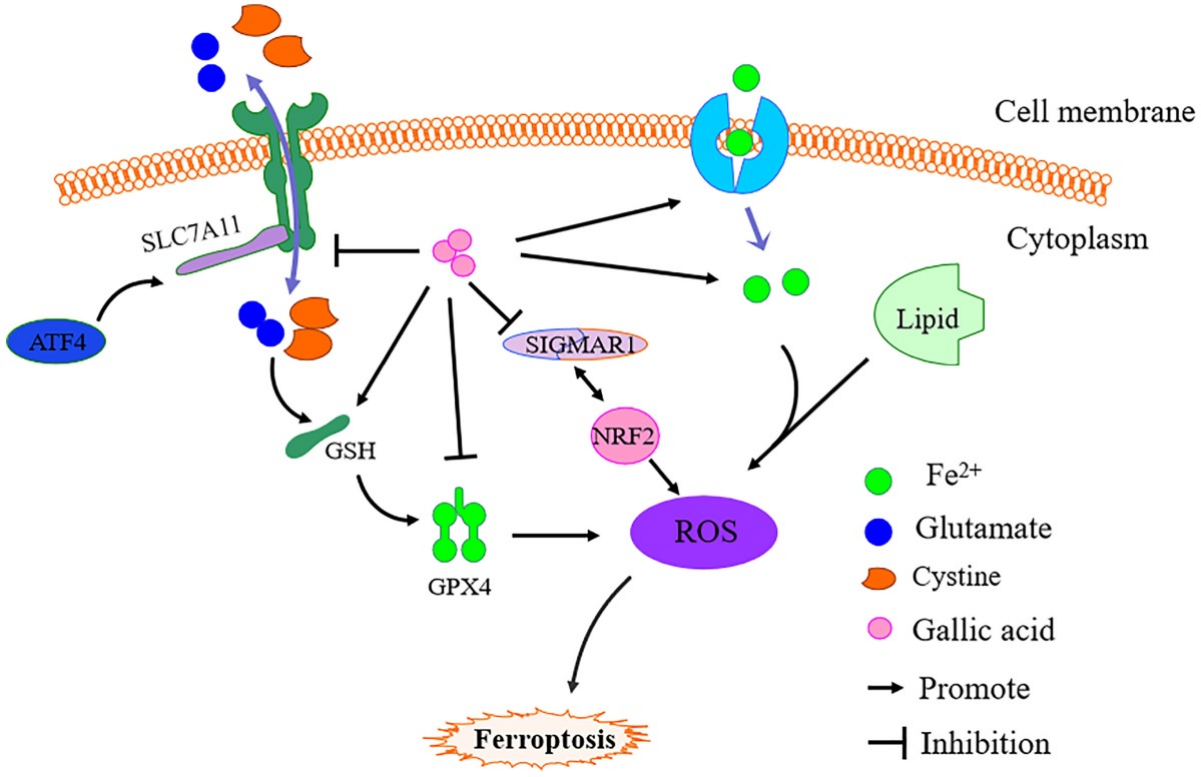
Simple schematic diagram of gallic acid regulating ferroptosis. In the presence of gallic acid, SLC7A11 is inhibited, and the transport balance of glutamate and cystine on the cell membrane is broken, resulting in the massive consumption of GSH and the production of ROS, thereby inhibiting the expression of GPX4. At the same time, gallic acid promotes the absorption of Fe^2+^ by cells, leading to Fe^2+^ overload in the cells, and the activation of SIGMAR1 promotes the further depletion of GSH and promotes the inhibition of GPX4.

Based on literature research and our prediction results, we further revealed the inhibitory mechanism of gallic acid on CRC. It is undeniable that under the action of gallic acid, GSH is consumed in large quantities, while Fe^2+^, MDA, and ROS have been accumulated. As we all know, ferroptosis is defined as an iron-dependent form of regulatory cell death. When the GSH-dependent lipid peroxide repair system is damaged, it occurs through the lethal accumulation of ROS, and lipid peroxidation is also the reason for promoting the production of ROS 32. The mechanism of ferroptosis has not yet been fully clarified. We simply mapped the regulatory mechanism of ferroptosis based on the available data (Figure 11). The main feature of iron-dependent death is the formation of specific lipid peroxides in the presence of catalytically active iron, which is regulated by the system xc-/GSH/GPX4 axis (endogenous offset), xCT (SLC7A11) ) is the specific subunit that constitutes system xc-. Once cystine is absorbed by system xc-, it is reduced to cysteine by GSH and/or thioredoxin reductase 1, and GSH and/or thioredoxin reductase 1 is used for GSH biosynthesis. GSH is a ubiquitous antioxidant in mammalian cells, and cysteine is the rate-limiting substrate in GSH biosynthesis. Therefore, hindering the production of cysteine in the cell will affect the GSH content and directly affect the activity of GPX4, so it is prone to ferroptosis 33. Simultaneously, GSH is the main cofactor for GPX4 to exert phospholipid peroxidase activity and catalyze the process of lipid peroxide reduction. Therefore, inhibition of SLC7A11 leads to depletion of GSH and subsequent inactivation of GPX4, which ultimately leads to the accumulation of ROS and ferroptosis. This point is consistent with the evidence we obtained in the investigation of the expression of GPX4 and SLC7A11.

It has been confirmed that genes related to iron metabolism are key mediators in the process of ferroptosis, such as transferrin (TF), transferrin receptor 1 (TFR1), ferritin heavy chain (FTH1), etc.. Fe^3+^ enters the cell through TFR1 and is finally reduced to Fe^2+^, and then ROS production is mediated through the fenton reaction. Increased TFR1 and down-regulation of FTH1 expression are the causes of iron overload in ferroptosis cells 34. Moreover, activated transcription factor 4 (ATF4) is an important mediator of endoplasmic reticulum stress. In addition to endoplasmic reticulum stress damage, a variety of microenvironmental stimuli (including exposure to ferroptosis inducers) can also cause it to increase. ATF4 can activate SLC7A11 and has recently been shown to play a negative regulatory role in ferroptosis. Therefore, the occurrence of ferroptosis may increase the expression of ATF4 due to cellular stress 35. Obviously, after the gallic acid treatment, the expression of TFR1 and ATF4 increased significantly. This shows that gallic acid affects the process of iron metabolism and the activation of ATF4.

Sigma-1 receptor (SIGMAR1) can participate in inhibiting the production of ROS by activating antioxidant response elements and degrading oxidized glutathione and glutamate. Evidence proves that the loss of SIGMAR1 function can block the expression of GPX4 *in vitro* and *in vivo* to induce ferroptosis in cancer cells 36. Although this regulatory pathway has not been fully elucidated, our research results show that SIGMAR1 showed a downward trend after gallic acid treatment.

Evidence has shown that TP53 antagonizes ferroptosis in CRC cells by favoring the localization of DPP4 toward a nuclear, enzymatically inactive pool 37. TP53 regulates ferroptosis through transcription or post-translational mechanisms, and exhibits an inhibitory effect on SLC7A11 38. Aurora kinase A (AURKA) is frequently overexpressed in several cancers. Inhibition of AURKA also leads to a significant reduction of GPX4, which indicates a possible link between AURKA and GPX4 39. The transcription factor nuclear factor erythroid 2-related factor 2 (NRF2) is a key regulator of the cellular antioxidant response, controlling the expression of genes that counteract oxidative and electrophilic stresses and ferroptosis can be inhibited by NRF2 through regulating SLC7A11 40-41. Therefore, the regulatory effects of TP53, AURKA, and NRF2 on ferroptosis may all be attributed to the effects on the expression of SLC7A11 and GPX4.

In summary, eight ferroptosis-related genes play a key role in CRC, and under the intervention of gallic acid, the expression of GPX4 and SLC7A11 decreased, and the expression of TFR1 increased. Extensive research also found that SIGMAR1 was inhibited and ATF4 was activated after the intervention of gallic acid. This shows that gallic acid is involved in the iron metabolism process and affects the ROS metabolism process, which in turn leads to ferroptosis. Nowadays, ferroptosis has played a significant role in the treatment of many diseases. As a ubiquitous and edible natural product, gallic acid can inhibit the progress of CRC by inducing ferroptosis. Even more surprising is that gallic acid seems to have the potential to improve the progression of UC. Various results show that gallic acid has great development value and application potential.

## 5. Conclusion

Ferroptosis is one of the cell regulation methods of CRC. AURKA, LPCAT3, TP53, CDKN2A, GPX4, PRNP, SLC7A11, and TFRC play a key role in CRC ferroptosis. In addition, with the combination of virtual docking prediction and experimental verification, we found that gallic acid can inhibit the growth of colon cancer cells, and its mechanism of action is related to ferroptosis. This provides new evidence for gallic acid to inhibit tumor growth and provides an experimental basis for gallic acid to become a prescription and beneficial dietary supplement.

## Supplementary Material

Supplementary figures and tables.

## Figures and Tables

**Table 1:** Docking details between the compound and the target proteins

Targets	PDB code	LiDock Score	Groups of comp.	Amino acid	Bonds name
GPX4	6hkq	72.39	OH	Glycine	Unfavorable Donor-Donor
			Benzene ring	Lysine	Pi-Alkyl
			OH	Lysine	Unfavorable Donor-Donor
			OH	Arginase	Conventional Hydrogen Bond
			OH	Alanine	Conventional Hydrogen Bond
			OH	Glycine	Carbon Hydrogen Bond
			Benzene ring	Isoleucine	Carbon Hydrogen Bond
TP53	3zme	75.53	OH	Cystine	Conventional Hydrogen Bond
			OH	Glutamic	Conventional Hydrogen Bond
			OH	Proline	Pi-Donor Hydrogen Bond
			Benzene ring	Glutamic	Pi-Anion
			OH	Threonine	Conventional Hydrogen Bond
			Benzene ring	Threonine	Carbon Hydrogen Bond
			Carbonyl	Threonine	Vander waals
TRFC	6y76	68.03	Benzene ring	Lysine	Pi-Alkyl
			OH	Tyrosine	conventional Hydrogen Bond
			OH	Lysine	Unfavorable Donor-Donor
			OH	Serine	Unfavorable Donor-Donor
			OH	Asparagine	Unfavorable Donor-Donor
			Carbonyl	Asparagine	Conventional Hydrogen Bond
AURKA	3p9j	66.87	OH	Phenylalanine	Unfavorable Donor-Donor
			Benzene ring	Aspartic	Pi-Anion
			OH	Aspartic	Conventional Hydrogen Bond
ATF4	1ci6	67.1	Benzene ring	Lysine	Pi-Alkyl
			Benzene ring	Alanine	Pi-Alkyl
			OH	Glutamine	Conventional Hydrogen Bond
			OH	Asparagine	Conventional Hydrogen Bond
			OH	Glutamine	Conventional Hydrogen Bond
NRF2	3zgc	69.2	OH	Histidine	Conventional Hydrogen Bond
			OH	Asparagine	Unfavorable Donor-Donor
			Benzene ring	Asparagine	Pi-Anion
			O	Asparagine	Conventional Hydrogen Bond
			O	Asparagine	Unfavorable Donor-Donor
SIGMAR1	6djz	86.4	Benzene ring	Arginine	Pi-Alkyl
			Benzene ring	Methionine	Pi-Sulfur
			Benzene ring	Tryptophan	Pi-Sulfur
			OH	Arginine	Conventional Hydrogen Bond
			OH	Arginine	Unfavorable Donor-Donor
CDKN2A	1dc2	N/A			
PRNP	3hjx	N/A			
