# Erratum: Ferroptosis-related Genes for Overall Survival Prediction in Patients with Colorectal Cancer can be Inhibited by Gallic acid

**DOI:** 10.7150/ijbs.68267

**Published:** 2021-11-06

**Authors:** Zongchao Hong, Peili Tang, Bo Liu, Chongwang Ran, Chong Yuan, Ying Zhang, Yi Lu, Xueyun Duan, Yanfang Yang, Hezhen Wu

**Affiliations:** 1 Faculty of Pharmacy, Hubei University of Chinese Medicine, Wuhan, China.; 2 Key Laboratory of Traditional Chinese Medicine Resources and Chemistry of Hubei Province, Wuhan, China.; 3 Collaborative Innovation Center of Traditional Chinese Medicine of New Products for Geriatrics Hubei Province, Wuhan, China.; 4 Hubei Provincial Hospital of Traditional Chinese Medicine, Wuhan, China.

In our paper 1, the sixth and seventh pictures in Figure 4 are incorrectly used. Figure 4 should be corrected as follows:

After the picture correction of SLC7A11 and TFRC in Fig.4, some descriptions in the original text are no longer accurate. The specific corrections are as follows:

1. In the “Abstract” section on page 942, line 12 is corrected to "......Six differentially expressed genes are related to overall survival.", line 14 is corrected to "...... Six new ferroptosis-related genes can be used" to predict the prognosis of CRC.”

2. On page 943, paragraph 3, line 13 is changed to "In addition, we also investigated the regulatory......".

3. Item 3.3 on page 946 of the “Results” section, lines 19 to 25 are corrected to "...the high expression of CDKN2A, GPX4, and PRNP are more likely to encounter CRC patients death earlier and shorten survival time (Figure 4 , p<0.05 means significant). A deeper understanding is that these 6 genes may provide insight into the prognosis of CRC. Moreover, Due to the important role of SLC7A11 and TFRC in the regulation of ferroptosis [25,34], and their pivotal position in the PPI network, we additionally included SLC7A11 and TFRC in the subsequent analysis. The differential expression of AURKA, LPCAT3, TP53, CDKN2A, GPX4, PRNP, SLC7A11, and TFRC has been further ......".

4. Item 3.4 on page 946 of the “Results” section, the first line is corrected to "As vital genes, AURKA, LPCAT3, TP53, CDKN2A, GPX4, PRNP, SLC7A11, and TFRC were used for GSEA analysis".

5. Item 3.5 on page 947 of the “Results” section, the third line is corrected to "...gallic acid with 8 vital targets".

6. In the first paragraph of the “Discussion” section on page 953, the third line is corrected to "......obtained 6 key genes that affect the overall survival of patients. In addition, SLC7A11 and TFRC, which play an important role in the regulation of ferroptosis, are also included in the analysis process. Among......".

References [25,34] are references in the original text, respectively:

[25] Liu T, Jiang L, Tavana O, Gu W. The Deubiquitylase OTUB1 Mediates Ferroptosis via Stabilization of SLC7A11. Cancer Res. 2019;79(8):1913-1924. doi:10.1158/0008-5472.CAN-18-3037

[34] Bogdan AR, Miyazawa M, Hashimoto K, et al. Regulators of Iron Homeostasis: New Players in Metabolism, Cell Death, and Disease. Trends Biochem Sci. 2016;41(3):274-286. doi:10.1016/j.tibs.2015.11.012

The authors regret these errors.

## Figures and Tables

**Figure 4:**
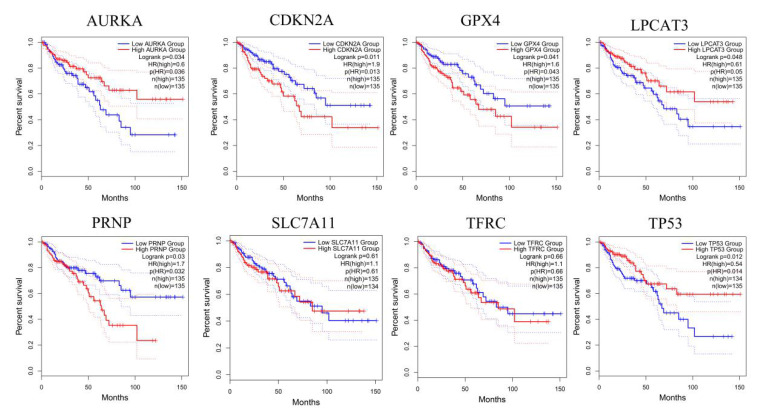
GEPIA analyzes the relationship between genes and the overall survival of CRC patients. The red line indicates the high expression group of genes and the blue line represents the low expression group of genes. AURKA, LPCAT3, TP53, CDKN2A, GPX4, and PRNP have a significant correlation with the overall survival of CRC patients. SLC7A11 and TFRC have no significant correlation with the overall survival of CRC patients.
