# Erratum for Ferroptosis-related Genes for Overall Survival Prediction in Patients with Colorectal Cancer can be Inhibited by Gallic acid

**DOI:** 10.7150/ijbs.69081

**Published:** 2022-01-21

**Authors:** Zongchao Hong, Peili Tang, Bo Liu, Chongwang Ran, Chong Yuan, Ying Zhang, Yi Lu, Xueyun Duan, Yanfang Yang, Hezhen Wu

**Affiliations:** 1 Faculty of Pharmacy, Hubei University of Chinese Medicine, Wuhan, China.; 2 Key Laboratory of Traditional Chinese Medicine Resources and Chemistry of Hubei Province, Wuhan, China.; 3 Collaborative Innovation Center of Traditional Chinese Medicine of New Products for Geriatrics Hubei Province, Wuhan, China.; 4 Hubei Provincial Hospital of Traditional Chinese Medicine, Wuhan, China.

In our paper 1, for the WB bands of HCT-116 cells in Figure 9, although they are from the same band, they belong to different lanes. However, due to lack of knowledge, we did not separate them with obvious marks. To avoid misleading, we need to replace them with WB strips in continuous lanes. Figure 9 should be corrected as follows:

After the picture correction in Fig.9, some descriptions in the original text are no longer accurate. The specific corrections are as follows:

1. In the “Abstract” section on page 942, line 12 is corrected to "...... adjacent normal tissues, and 23 of them are identified in the GEPIA database. Among them...... ".

2. We found a small error on page 944, paragraph 6, line 6, which needs to be corrected as "......TFR1 antibody (#bsm-52793R)......".

3. Item 3.3 on page 946 of the “Results” section, line 6 is corrected to "... in CRC. Among them, GPX4, PRNP, TFRC, and TP53 used "Match TCGA normal and GTEx data" in the GEPIA analysis, and "Match TCGA normal data" was used in the analysis of the remaining 19 genes. According to ......".

4. Item 3.6 on page 947 of the “Results” section, line 5 is corrected to "... effect, so unless otherwise specified, this concentration was used for ......".

5. Item 3.8 on page 950 of the “Results” section, lines 5 to 10 need to be corrected as "......WB experiments. After treating HCT-116 cells with different doses of gallic acid, the expression of GPX4 and SCL7A11 were inhibited, while the expression of TFR1 increased. The ferroptosis inhibitor......". Line 17 is corrected to “......these trends. Not surprisingly, after we treated Caco-2 cells with 0.2 mM gallic acid, the expression trend of these proteins was verified. These results......”.

6. In the legend of Figure 9 in page 952, we modified it to: “......reversed the expression trend of these proteins. HCT-116 cells were treated with low-dose (0.2 mM) and high-dose (0.4 mM) gallic acid. Caco-2 cells were treated with 0.2 mM gallic acid......”.

The revised Figure 9 and the added description will not affect the overall structure and conclusion of this article.

The authors regret these errors.

## Figures and Tables

**Figure 9:**
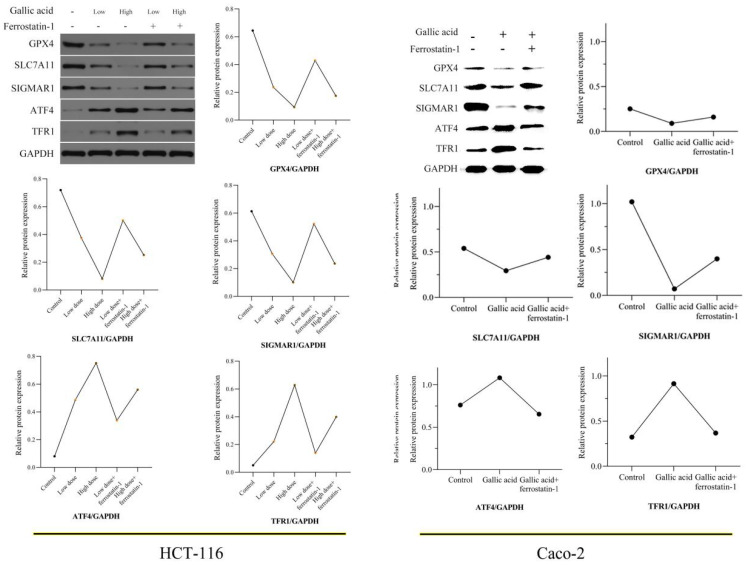
Gallic acid affects the expression of ferroptosis-related proteins. GAPDH serves as an internal reference protein. The relative expression levels of GPX4, SLC7A11, and SIGMAR1 decreased, while the relative expression levels of ATF4 and TFR1 increased. Ferrostatin-1 as a ferroptosis inhibitor reversed the expression trend of these proteins. HCT-116 cells were treated with low-dose (0.2 mM) and high-dose (0.4 mM) gallic acid. Caco-2 cells were treated with 0.2 mM gallic acid.
